# 

**Published:** 1824-04

**Authors:** 


### MONTHLY CATALOGUE OF MEDICAL BOOKS.

*** *It having been hinted to us that gentlemen, sending copies of their Works, prefer having their titles given at length, it is our intention, in future, to comply with this suggestion: but it is to be observed, that no books can be entered on this List except those sent to us for the purpose;—finding, in the lists hitherto transmitted, that the names of works have frequently been given as published, which have not appeared for weeks, or even months after.*

An Enquiry into the Causes of the Curvature of the Spine, with Suggestions as to the best Means of preventing, or, when formed, of removing, Lateral Curvature. By T. JARROLD, M.D.—pp. 147. Longman and Co. 1823.

An Essay on the Blood: comprehending the chief Circumstances which influence its Coagulation; the Nature of the Buffy Coat; with a concise Medical View of the State of the Blood in Disease, and an Account of the Powers of a saturated Solution of Alum as a Styptic Remedy in Hemorrhage. By CHARLES SCUDAMORE, M.D. F.R.S. Member of the College of Physicians in London; Honorary Member of Trinity College, Dublin, of the Medico-Chirurgical Society of Edinburgh, and of the Medical Society of Paris; Member of the Medico-Chirurgical Society of London; Physician in ordinary to H. R. H. the Prince Leopold of Saxe-Coburg, &c. &c.—pp. 162. Longman, London, 1824.

Pathological Observations, Part I. on Dropsy, Purpurea, and the Influenza of the latter end of the year 1822 and beginning of that of 1823; and particularly on the morbid Changes of the Blood, and their Influence on the Production and Cause of these Diseases, illustrated by select Cases and Dissections. By W. Stoker, M.D. Edinburgh; Licentiate of the King and Queen's College of Physicians in Ireland; Senior Physician to the Fever Hospital and House of Recovery, &c. &c.—pp. 244. Dublin, 1823.

The Principles of Forensic Medicine systematically arranged, and applied to British Practice. By JOHN GORDON SMITH, M.D. Lecturer on Political Medicine. Second Edition, greatly enlarged.—pp. 569. Underwood, London, 1824.

A Letter from GOLDSWORTHY GURNEY, author of "Lectures on Chemical Science," to W. T. BRANDE, Esq. Professor of Chemistry at the Royal Institution, in a late Review of Mr. Gurney's Work in the "Quarterly Journal of Science, Literature, and the Arts."—pp. 22. London, 1824.

Nugæ Chirurgicæ; or, a Biographical Miscellany, illustrative of a Collection of Professional Portraits. By WM. WADD, Esq. F.L.S. Surgeon Extraordinary to the King; Fellow of the Royal College of Surgeons, London, and of the Societé de Medecine, Paris.—pp. 276. London, 1824.

Practical Observations on the Removal of every Species and Variety of Cataract by Hyalonyxis, or Vitreous Operation, illustrated by Cases: with critical and general Remarks on the other Methods employed. By JOHN BOWEN, M.D. Fellow of the Royal College of Physicians, Edinburgh; Member of the Royal College of Surgeons, London; Member of the Fiso-critico of Science, and of the Arcadia of Rome; corresponding Member of the Georgofili of Florence, &c. &c. —pp. 120. London, 1824. *(This book was accidentally omitted in our previous list.)*

Observations on Acute Rheumatism, and its Metastasis to the Heart, &c. By THOMAS COX, M.D. Fellow of the Medical Society of London; Member of the Royal Medical Society of Edinburgh; and honorary Member of the Physical Society, Guy's Hospital.—pp. 65. London, 1824.

Symptomatology; or, the Art of detecting Disease: a Lecture, occasionally read to the Pupils at the Westminster Hospital, and published according to their request. By ALEX. P. BUCHAN, M.D. F.L.S. late senior Physician to that Institution, and Member of the Royal College of Physicians, London. To which are added, Tables of Symptoms.—pp. 190. London, 1824.

A Second Letter to Sir J. COX HIPPISLEY, Bart. on the Mischiefs incidental to the Tread-Wheel as an Instrument of Prison Discipline; containing an Examination of the official Reports upon this subject, returned to the Secretary of State's Office during the present Session of Parliament. By J. MASON GOOD, M.D. F.R.S. &c. &c.—pp. 75. Baldwin, Cradock, and Joy, 1824.

A Letter on the Nature and Effects of the Tread-Wheel, as an Instrument of Prison Labour and Punishment, addressed to the Right Hon. ROBERT PEEL, M.P. his Majesty's principal Secretary of State for the Home Department, &c. &c. With an Appendix of Notes and Cases. By one of his Constituents, and a Magistrate of the county of Surrey.—pp. 174. Hatchard and Son, London, 1824.

Thoughts on Prison-Labour, &c. &c. By a Student of the Inner Temple.—pp. 132, and Appendix, pp. cccxlviii. Rodwell and Martin, London, 1824.

Leçons sur les Epidemies et l'Hygiene publique. Par F. E. FODERE. Tom. III.—Paris, 1824.

---

Plate I. Section of the Leg; forming part of a Series of Engravings designed as practical Illustrations of the Surgical Anatomy of the Blood-vessels, Nerves, &c. relating to Amputation. By THOMAS ALCOCK, Surgeon. Proof impression, coloured under the Author's immediate direction.

The object of these engravings, we are informed by Mr. Alcock, "is to afford the surgeon the same facilities in distinguishing, by relative position, whatever it is essential he should readily find or certainly avoid, as the mariner experiences by consulting his chart."

The first plate of this intended series represents a transverse section of the right leg, a little below the knee, the face of the stump being turned forwards, as when the operator is about to secure the arteries, the limb being fore-shortened. There is likewise an outline, with copious references, explanatory of the finished engraving. The design of these plates appears to us extremely good, and well calculated to communicate a distinct and practical acquaintance with the relative position of the parts; and we anticipate that they will meet with a favourable reception, particularly from the junior part of the profession, whom they are particularly, but not exclusively, calculated to benefit.

